# Initial Spore Density Has an Influence on Ochratoxin A Content in *Aspergillus ochraceus* CGMCC 3.4412 in PDB and Its Interaction with Seeds

**DOI:** 10.3390/toxins9040146

**Published:** 2017-04-21

**Authors:** Caiyan Li, Yanmin Song, Lu Xiong, Kunlun Huang, Zhihong Liang

**Affiliations:** 1 Beijing Advanced Innovation Center for Food Nutrition and Human Health, College of Food Science and Nutritional Engineering, China Agricultural University, Beijing 100083, China; lcy9048@163.com (C.L.); songyanminyx@sina.com (Y.S.); lxxionglu@163.com (L.X.); hkl009@163.com (K.H.); 2 The Supervision, Inspection and Testing Center of Genetically Modified Organisms, Ministry of Agriculture, Beijing 100083, China

**Keywords:** quorum sensing, density effect, Ochratoxin A, fatty acids, oxidative stress

## Abstract

The morphology and secondary metabolism of *Aspergillus* spp. are associated with initial spore density (ISD). Fatty acids (FA) are involved in the biosynthesis of aflatoxins (AF) through *Aspergillus* quorum sensing (QS). Here, we studied how ochratoxin A (OTA) was regulated by spore density in *Aspergillus ochraceus* CGMCC 3.4412. The results contribute to understanding the role of spore density in morphogenesis, OTA biosynthesis, and host–pathogen interactions. When *A. ochraceus* was grown in Potato Dextrose Broth (PDB) media at different spore densities (from 10^1^ to 10^6^ spores/mL), more OTA was produced when ISD were increased, but a higher level of ISD inhibited OTA biosynthesis. Seed infection studies showed that peanuts (*Arachis hypogaea*) and soybeans (*Glycine max*) with high FA content were more susceptible to OTA production when infected by *A. ochraceus* and reactive oxygen species (ROS)-induced OTA biosynthesis. These results suggested that FA was vital for OTA biosynthesis, and that oxidative stress was closely related to OTA biosynthesis in *A. ochraceus*.

## 1. Introduction

Ochratoxin A (OTA) is a mycotoxin found in a variety of agricultural commodities, which include cereal grains and dried fruits. It is produced mainly by *A. ochraceus*, *A. carbonarius*, and *A. niger* [1]. OTA has been shown to be carcinogenic in animals. The kidney is the main target organ [2,3]. It has been recognized as a Group 2B carcinogen by International Agency for Research on Cancer Collection Center (IARC) [4], although OTA-related carcinogenicity has not been determined conclusively to occur in humans [5,6].

Among the OTA-producing fungi, *A. ochraceus* is the most common in tropical regions [7], and *Penicillium verrucosum* predominates in temperate regions [8]. Other OTA producing strains include *A. carbonarius* and *A. alliaceus* [9,10].

The OTA biosynthetic pathway has not yet been determined in detail [11], although the process of OTA biosynthesis has been analyzed [12]. These studies show that the first steps involve the pentaketide pathway [13]. The same study indicated that chlorination was a penultimate biosynthetic step in OTA biosynthesis [14]. The modulating factors that are involved in OTA biosynthesis are still not totally clear. The genes *pks*, *p450-B03*, and *p450-H11* are coregulated during OTA biosynthesis, and they are likely to be engaged in a cluster that presides over a multistep enzymatic reaction [15].

Quorum sensing (QS) is a density-dependent process that can regulate several behaviors in bacteria, such as secretion of virulence factors, biofilm formation, competence, and bioluminescence, and the proliferation and secondary metabolism of fungi are regulated by QS [16,17]. There is very little information on QS in filamentous fungi [18]. However, *A. flavus* reproduces in a density-dependent manner [17,19]. As the population transitions to a high cell density, the reverse phenotype is observed to have reduced sclerotia production and increased conidiation. In addition to development, secondary metabolism is also regulated in a density-dependent manner, where the pattern of aflatoxin (AF) biosynthesis mirrors that of sclerotia production: much higher production occurs at low population density [19,20].

Several molecules and genes have been found to be important in QS of *A. flavus*. Chief among these are oxylipins, a group of oxygenated polyunsaturated fatty acids, which are molecular signals across the fungal, plant and animal kingdoms. Fungal oxylipins originated from oleic acid (18:1), linoleic acid (18:2), and linolenic acid (18:3) after an O_2_ molecule is added to polyunsaturated fatty acids [21]. Lipoperoxidative signalling is essential for the regulation of mycotoxin biosynthesis, conidiogenesis, and sclerotia formation in *A. nidulans*, *A. flavus* and *A. Parasiticus* [15].

Oxidative stress is also regarded as a trigger of many metabolites in all organisms; previous studies showed a direct link between oxidative stress and AF formation [22]. Reactive oxygen species (ROS) can be observed when cells start to metabolize, and overproduction leads to the action of oxidative stressors that are present in the environment [23]. To eliminate the underlying serious assemblage of ROS, cells form enzymatic and nonenzymatic systems. To remove the ROS, cells require the involvement of superoxide dismutase (SOD) and catalase (CAT), which are the most important antioxidant enzymes [22]. Collectively referred to as precocious sexual inducer (PSI) factors in *Aspergillus* spp., oxylipins are known to regulate the balance between asexual and sexual development in *Aspergillus* spp. including *A. flavus* [24].

In this study, our objective was to do a preliminary research of quorum sensing in *A. ochraceus*, which included how spore densities affected morphology, OTA production, and the expression of genes that were involved in OTA biosynthesis. We found that OTA was regulated by density-dependent mechanisms, and that, after increasing the cell density (from 10^1^ to 10^3^ spores/mL), more OTA was produced. However, a higher concentration of ISD (from 10^4^ to 10^6^ spores/mL) inhibited OTA biosynthesis, which suggested that lipid modifiers have an effect on these density-dependent phenomena. Seed infection studies showed that peanuts and soybeans with high fatty acids (FA) content were more susceptible to OTA accumulation when infected by *A. ochraceus* and ROS-induced OTA biosynthesis. 

## 2. Results

### 2.1. Spore Density-Dependent OTA Production in Potato Dextrose Broth (PDB) Media

**Figure 1:**
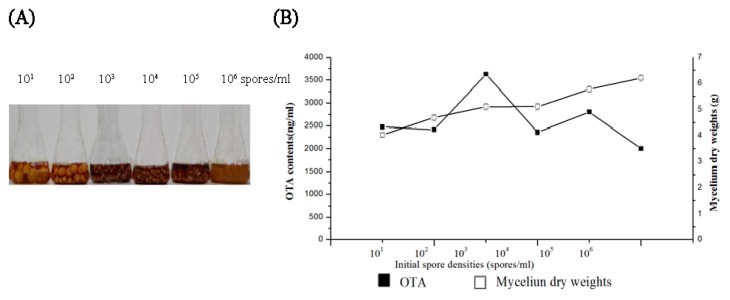
Spore density-dependent phenomenon in morphological transitions and ochratoxin A (OTA) production in *A. ochraceus* in potato dextrose broth (PDB) media. (**A**) images show the density-dependent phenomena after six days of incubation; (**B**) mycelial growth and OTA production by *A. ochraceus* CGMCC 3.4412 that was cultured in PDB media on a shaker (180 rpm) at 28 °C in the dark for six days with initial spore density (ISD) of 10^1^, 10^2^, 10^3^, 10^4^, 10^5^, and 10^6^ spores/mL. The mycelium dry weights were measured after six days. All results were the mean ± standard deviation (SD) of three measurements mixed from three independent samples.

To investigate if the ISD affects development and OTA production in *A. ochraceus*, 10^1^, 10^2^, 10^3^, 10^4^, 10^5^, and 10^6^ spores/mL were inoculated into PDB media. Increasing the spore density (from 10^1^ to 10^6^ spores/mL) led to the formation of different sizes of mycelial pellets. Cultures grown with low cell densities (10^1^, 10^2^, and 10^3^ spores/mL) produced fewer, but larger mycelial pellets than using high spore densities (10^5^ and 10^6^ spores/mL) (Figure 1A). The phenomenon suggested that there is a link between proliferation of biomass and density in *A. ochraceus*. 

We observed an OTA production, density-dependent phenomenon. When the ISD increased progressively (from 10^1^ to 10^3^ spores/mL), an increasing amount of OTA was detected in PDB media after six days. OTA production reached the maximum level when cultured at the ISD of 10^3^ spores/mL, and then OTA decreased significantly. At 10^4^ spores/mL, OTA production was reduced significantly compared to production of 10^3^ spores/mL (Figure 1B), which suggested inhibition of OTA production in the high-density PBD culture. Growth that is accelerated in PDB media with large ISD may result in a reduction of intracellular oxygen, and then a decrease in oxidative stress. The less oxidative stress finally resisted the OTA production. In addition, mycelium dry weights increased continuously when ISD was increased from 10^1^ to 10^6^ spores/mL (Figure 1B), which showed that the growth in the high-density PDB culture was not be inhibited. 

### 2.2. Influence of Spore Densities on Transcript Levels of OTA Biosynthesis-Related Genes

**Figure 2:**
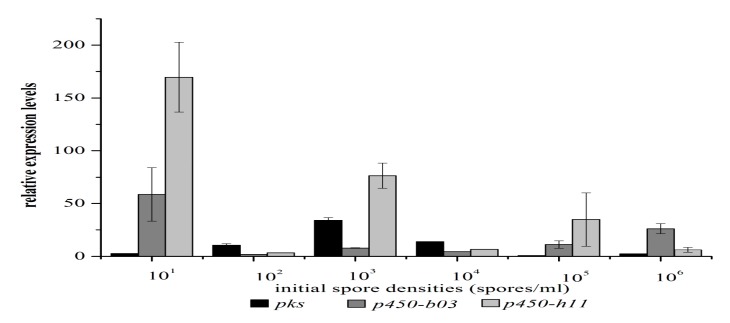
High ISD repressed the expressions of OTA biosynthesis genes in *A. ochraceus*. qRT-PCR (quantitative reverse transcription PCR) was used to analyze expressions of OTA biosynthesis genes (*pks*, *p450-b03*, *p450-h11*) by *A. ochraceus* CGMCC 3.4412 cultured in PDB media for six days with ISD of 10^1^, 10^2^, 10^3^, 10^4^, 10^5^, and 10^6^ spores/mL. The relative expressions were quantified by the expression level of the Ao18s gene. Data are the means of three separate experiments.

To determine how ISD affects OTA production, expression of OTA biosynthesis-related genes were examined by quantitative reverse transcription PCR (qRT-PCR) in mycelia that were initiated with 10^1^, 10^2^, 10^3^, 10^4^, 10^5^, and 10^6^ spores/mL at 28 °C for six days. In *A. ochraceus*, the *pks* transcript level increased at first (Figure 2), reached a peak of 10^3^ spores/mL, and then descended while the spore density increased (Figure 1B); the accumulation was about 2.5-fold higher at 10^3^spores/mL than at 10^4^spores/mL.

The transcription level of *p450-H11* and *p450-B03* genes both achieved peaks at 10^1^ spores/mL, and then decreased, which did not mirror closely that of the *pks* gene under the same growth conditions; this indicated their non-involvement with *pks* in OTA biosynthesis in this process and that they have a different manner of regulation. Nevertheless, we noticed that cultures with a high initial density restrained the expression of both *p450-H11* and *p450-B03* genes simultaneously. Both genes also can be regulated during growth at different spore densities when the ISD increased gradually (from 10^2^ to 10^6^ spores/mL); *pks* gene transcription behaved in a similar fashion. However, the highest levels were observed at 10^1^ spores/mL, where *p450-H11* and *p450-B03* transcript levels were 2.2- and 7.3-fold higher than at 10^3^ spores/mL.

### 2.3. Oxylipin-Mediated Host-Fungus Cross Talk

**Figure 3:**
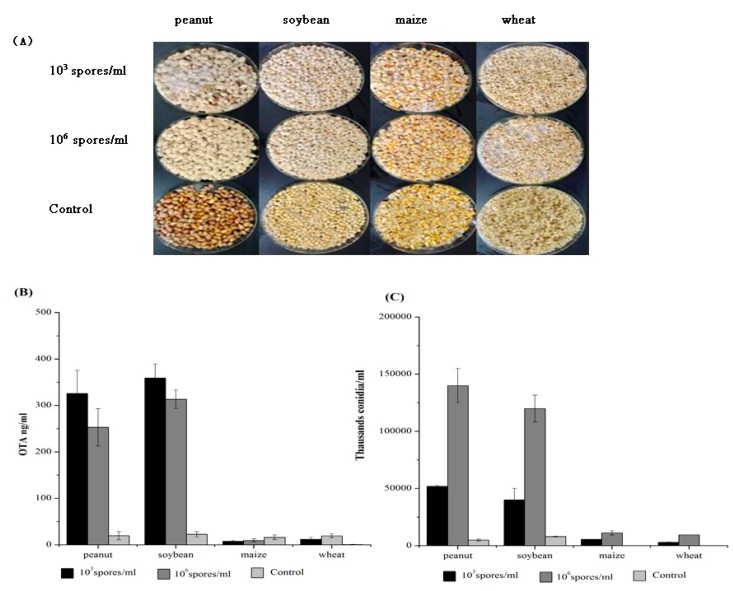
(**A**) growth on seeds; (**B**) OTA production on seeds. Colonization of seeds as reflected by conidiation levels: Wild type CGMCC 3.4412 was grown on seeds for six days at 28 °C; (**C**) conidia were counted from three replicates that contained 10 seeds each.

**Figure 4:**
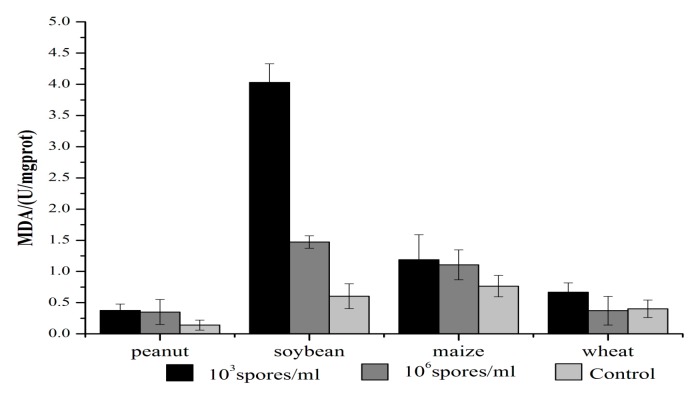
Effect of ISD on lipid peroxidation in seeds infected by *A. ochraceus*.

We examined the capacity of *A. ochraceus* to produce OTA in live seeds. Here, we assessed the effect of spore density on OTA production. Surface-sterilized seeds were infected with suspensions of 10^3^ spores/mL and 10^6^ spores/mL of *A. ochraceus* at the same time. It was evident that seeds of peanuts and soybeans, which have a high oil content, were more susceptible to infection by *A. ochraceus* compared with maize(Zea mays) and wheat(Triticum) because the surface color of peanuts and soybeans change more significantly (Figure 3A). In addition, OTA biosynthesis was significantly higher in peanuts and soybeans than in maize and wheat (Figure 3B, at 10^3^ spores/mL, peanut = 326 ng/mL; soybean = 359 ng/mL; maize = 8 ng/mL; wheat = 11 ng/mL). At 10^3^ spores/mL, OTA production was much greater compared to seeds with 10^6^ spores/mL, which suggested that OTA production was inhibited in seeds with a high density of spores. Conidial numbers increased with increasing cell density. ISD of seeds with 10^3^ spores/mL consistently produced fewer conidia than the ISD of seeds at 10^6^ spores/mL in both peanuts and corn seeds (Figure 3C). Conidial production with ISD of 10^3^ spores/mL decreased 63% and 67% compared with seeds at 10^6^ spores/mL on peanuts and soybeans, respectively (*p* ≤ 0.05). However, the levels of conidiation and the amount of OTA biosynthesis were low on maize and wheat, regardless of whether the ISD was 10^3^ spores/mL or 10^6^ spores/mL (Figure 4B). It may be that the *A. ochraceus* CGMCC 3.4412 strain has lost the density-sensing ability in maize and wheat.

### 2.4. Effects of A. ochraceus Infection on MDA

Lipid peroxidation was estimated by measuring malonic dialdehyde (MDA) levels (Figure 4). MDA levels were higher in soybeans than in peanuts, maize, or wheat. In soybeans, the formation of MDA was 4.0 U/mg protein at 10^3^ spores/mL and 1.5 U/mg protein at 10^6^ spores/mL. In contrast, there was less MDA formed in peanuts maize and wheat, and there was a small reduction in MDA formation with ISD of 10^6^ spores/mL compared with 10^3^ spores/mL in peanuts, maize, and wheat.

### 2.5. Effects of A. ochraceus Infection on ROS Activity and Antioxidant Enzymes

**Figure 5:**
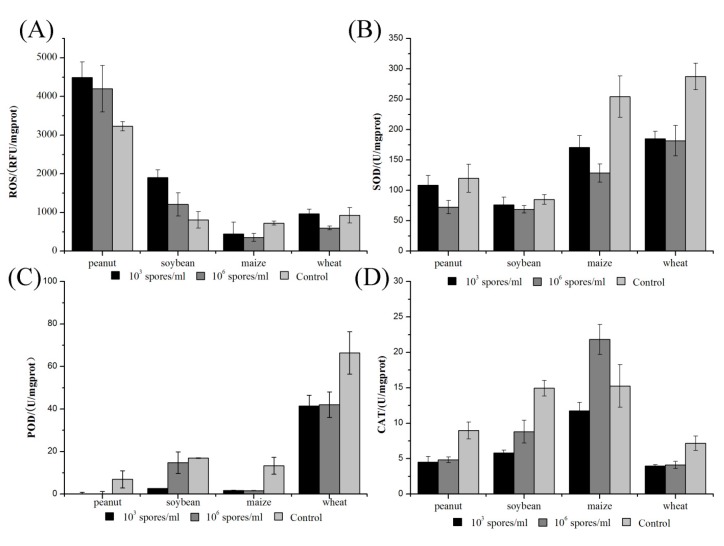
(**A**) The accumulation of reactive oxygen species (ROS) in seeds after being infected by *A. ochraceus*. ROS content was measured by H2DCFDA fluorescence; (**B**) the activity of antioxidant enzyme superoxide dismutase (SOD) (U/mg protein) at different times of incubation in seeds infected by *A. ochraceus*; (**C**) the activity of antioxidant enzyme peroxidase (POD) (U/mg protein) at different times of incubation in seeds infected by *A. ochraceus* and (**D**) the activity of antioxidant enzyme catalase (CAT) (U/mg protein) at different times of incubation in seeds infected by *A. ochraceus.* The results are the mean ± SD of three determinations from three separate experiments.

*A. ochraceus* induced ROS accumulation, which was determined quantitatively with dichloro-dihydrofluorescein diacetate (H2DCFDA), and the results suggested that *A. ochraceus* infection led to a continuous and significant increase in ROS content. The accumulation of ROS in peanuts, soybeans, maize, and wheat was increased compared to the control after being infected by *A. ochraceus*, and the ROS content at 10^3^ spores/mL was higher than that at 10^6^ spores/mL in peanuts and soybeans. On the contrary, the ROS content in maize and wheat decreased, and there was no significant difference between ROS content at 10^3^ spores/mL and 10^6^ spores/mL. In wheat, the ROS content of 10^3^ spores/mL did not change significantly, and it decreased with ISD at 10^6^ spores/ mL (Figure 5A).

The activity of the antioxidant enzymes SOD, CAT, and peroxidase (POD) in seeds of peanuts, soybeans, maize, and wheat decreased significantly after six days of incubation with *A. ochraceus* (Figure 5B–D). The activity of CAT, SOD, and POD all significantly decreased in infected seeds compared to activity in the control. These antioxidant enzymes displayed significantly lower activity than under control conditions. However, the activity of CAT enzymes was higher than that in control with ISD of 10^6^ spores/mL in maize. POD activity was not significantly different at 10^3^ spores/mL compared to those detected at 10^6^ spores/mL. SOD activity at 10^6^ spores/mL then decreased to levels lower than that observed at 10^3^ spores/mL in peanuts and soybeans. However, the activity of CAT showed no significant changes. In peanuts and soybeans, the reverse trend was observed, with higher activity at 10^6^ spores/mL than at 10^3^ spores/mL. 

## 3. Discussion

**Figure 6:**
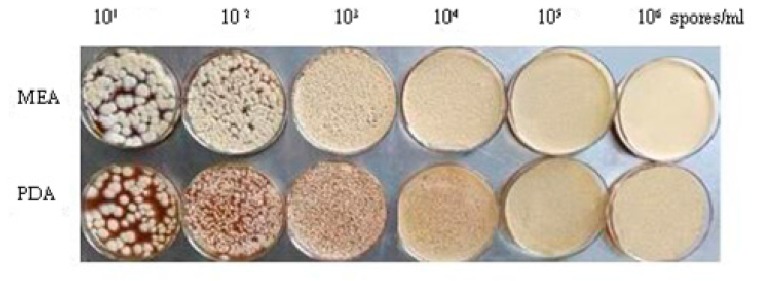
Morphology of *A. ochraceus* under different spore densities in malt extract agar (MEA) and potato dextrose agar (PDA).

OTA was more productive when ISD increased, although higher densities of ISD restrained OTA biosynthesis. This phenomenon was also observed in potato dextrose agar (PDA) and malt extract agar (MEA) media. QS is a density-dependent phenomenon that results in a coordinated response from the population [20]. *A. flavus* reproduces in a density-dependent manner where low population densities are characterized by increased sclerotia production and reduced conidiation. As the population transitions to high cell density, the reverse phenotype is observed with reduced sclerotia production and increased conidiation [19,20]. However, the phenomenon of morphological transformation from sclerotia to conidiation was not observed when *A. ochraceus* was cultured in PDA and MEA (Figure 6). Sclerotia production might be related to culture media. The phenomenon was observed when *A. flavus* was cultured in glucose minimal medium (AOBOX, Beijing, China). Based on a previous study, the transcription of *p450-H11* and *p450-B03* genes mirrored closely that of the *pks* gene under the same conditions, which suggested the involvement of the abovementioned three genes in OTA biosynthesis [15], but in our experiments, the two genes did not closely mirror that of the *pks* gene in this process. It is probable that more genes that are involved in a density-dependent will be discovered.

Studies of the interactions of *A. ochraceus* with seeds have also implicated the role of fatty acids and oxidative stress on OTA production. In this study, we demonstrated that seeds enriched in FA, such as peanuts and soybeans, were more susceptible to OTA accumulation when infected by *A. ochraceus*. It is generally known that seeds of plants such as maize, peanuts, and walnuts that are rich in oils are more susceptible to AF production when infected by AF-producing *Aspergillus* spp. [25]. FA is essential for AF biosynthesis [26]. Here, we proved that FA is also vital for OTA biosynthesis. 

Oxidative stress is also a trigger of many metabolites in organisms. ROS is important in fungal development [27,28]. In *A. parasiticus*, ROS can control sclerotium formation [29]. In *A. flavus*, the biosynthesis of mycotoxins has a close connection with various stages of fungal development [30], and oxidative stress is essential in AF production [31]. Our results indicated that oxidative stress and OTA production in *A. ochraceus* were linked tightly. We noticed that increased levels of ROS had a close connection with increased levels of OTA biosynthesis in *A. ochraceus* when it infected different seeds. Further investigations of how ROS triggers OTA production may help to control the biosynthesis of mycotoxins.

## 4. Conclusions

The results of this study suggested that when *A. ochraceus* was grown in PDB at different spore densities (from 10^1^ to 10^6^ cells/mL), OTA production was more when ISD increased, but higher densities inhibited OTA biosynthesis. Our study of fungal infection in seeds showed that peanuts and soybeans that are rich in FA are more susceptible to OTA accumulation when infected by *A. ochraceus*. These results indicated that FA was important for OTA biosynthesis. Meanwhile, oxidative stress and OTA production in *A. ochraceus* were linked tightly.OTA produced by *A. ochraceus* may be related to FA and oxidative stress. Therefore, antioxidants could prevent the production of OTA in agricultural commodities. Understanding the common mechanisms of biosynthesis in different mycotoxins would promote the development of suitable strategies to control several mycotoxins simultaneously.

## 5. Materials and Methods

### 5.1. Fungal Strain and Culture Conditions

*A. ochraceus* strain CGMCC 3.4412 was purchased from the Institute of Microbiology, Chinese Academy of Sciences, Beijing, China. The strain was cultured on PDA medium (AOBOX, Beijing, China) and incubated at 28 °C. To exam OTA production, *A. ochraceus* CGMCC 3.4412 was inoculated and cultured on PDA at 28 °C in dark conditions for 6 days to obtain a heavy sporulation culture. The spores were resuspended in sterile distilled water that contained 0.05% Tween 20. Spore concentration was counted using a haemacytometer, and the final concentration was adjusted to 10^7^ spores/mL. The spore suspensions were diluted when necessary. Spore suspensions (5 mL) were kept in 45 mL Potato Dextrose Broth (AOBOX, Beijing, China) media, and the mixture was shaken (180 rpm) at 28 °C in the dark.

### 5.2. Determinations of Fungal Dry Weights and OTA Content

To determine dry weights, mycelia grown in 50 mL media were harvested by two layers of filter paper, and then washed by sterilized water; before obtaining a constant weight, mycelia also should be freeze-dried. For extraction of OTA from media, 1 mL of medium from each flask were extracted with an equal volume of chloroform. The chloroform fraction was evaporated under nitrogen. The residues were redissolved in 1 mL methanol. Samples were stored at −80 °C until analyzed quantitatively for mycotoxins by an ELISA kit.

### 5.3. Extraction of RNA and Preparation of cDNA

Mycelia were harvested and frozen immediately at −80 °C. To extract the RNA, the mycelia were ground in liquid nitrogen in a pre-cooled mortar and pestle.

Total RNA was extracted from the ground mycelia using an RNeasy Plant kit. cDNA was prepared using cDNA Quantscript RT kit. 

### 5.4. qRT-PCR Analysis of Gene Transcript Accumulation

qRT-PCR was conducted in a Bio-Rad C1000TM Thermal Cycler (Hercules, CA, USA) installed with CFX96TM software (CFX96 system, Bio-Rad, Hercules, CA, USA) using the primers listed in Table 1 and replicated three times in a 25 μL reaction volume. Real Master Mix (SYBR green) was used.

The PCR amplification program was as follows: one cycle of 95 °C for 10 min followed by 39 cycles of denaturation (15 s at 95 °C), annealing (15 s at 58 °C), and extension (30 s at 72 °C), with a final elongation of 72 °C for 1 min. The amplification was followed by a melting curve analysis program with a temperature range of 65–95 °C. 18S ribosomal RNA was used an endogenous control.

The gene expression values were normalized to those of reference gene Ao18s. The relative gene expression was evaluated using the 2^−ΔΔCT^ method, where ΔΔCT = (CT, Target − CT, Ao18s) Infected −(CT, Target − CT, Ao18s) Control.

### 5.5. Peanut, Bean, Wheat and Maize Seed Infection Studies and OTA Analysis from Seeds

Infection studies and OTA analysis of peanuts, soybeans, wheat, and maize seeds were carried out as described previously [17]. First, seeds were surface sterilized by dipping them in a beaker that contained 0.05% sodium hypochlorite in sterile water for 3 min. Then, seeds were washed by sterile distilled water, 70% ethanol, and sterile distilled water again. This was followed by complete drainage, and then the seeds were placed in a Petri dish until the time of infection. All steps were performed aseptically in a biosafety hood.

For the infection experiment, 200 g of seeds was immersed in 200 mL of sterile distilled water (control) or sterile distilled water with fungal conidia in a 500 mL flask while shaking for 30 min at 200 rpm. Seeds were placed in Petri dishes that were lined with moist filter paper to maintain high humidity and incubated for 6 days at 28 °C in dark conditions. Then, 200 g of seeds per replicate was transferred to a humidity chamber and incubated as described previously. Seeds were collected in tubes with the addition of 3 mL of 0.01% Tween 80 (vol/vol, water) after 6 days, and vortexed vigorously. One milliliter was removed from each sample, and 3 mL of chloroform was added, followed by shaking for 10 min at 200 rpm. Samples were allowed to stand for an additional 10 min at room temperature, vortexed briefly, and centrifuged for 5 min at 8000 rpm to collect the organic lower phase. The chloroform extract was evaporated to dryness under nitrogen. The residues were redissolved in 1 mL methanol.

### 5.6. Measurement of ROS, MDA, and Antioxidant Enzymes

The ROS content was measured by H2DCFDA fluorescence, according to the ROS kit. The ROS kit and MAD, SOD, POD, CAT were all purchased from Nanjing Jiancheng Bioengineering Institute, Nanjing, China [32], in relation to MDA, SOD, POD, CAT.

## Figures and Tables

**Table 1:** PCR primers used in this study.

Primer	Sequence	Annealing Temperature	Reference
Pks-F	TTCTCTGCGCTTCTCACATC	60 °C	[11]
Pks-R	AACATCATAAGAGGTCAACA	60 °C	[11]
P450-B03-F	CTCGGTGACATCAGGGGTATC	60 °C	[11]
P450-B03-R	AGCGTATTCAGTCACTCATTCAGA	60 °C	[11]
P450-H11-F	AGAACGGGATGCCAAAACAGTGAG	64 °C	[11]
P450-H11-R	AAGAATGCGAGGGATGGGATAACC	64 °C	[11]
Ao-18s-F	ATGGCCGTTCTTAGTTGGTG	60 °C	[15]
Ao-18s-R	GTACAAAGGGCAGGGACGTA	60 °C	[15]

## Abbreviations

The following abbreviations are used in this manuscript:

ISD	Initial spore density
FA	Fatty acids
AF	Aflatoxin
QS	Quorum sensing
OTA	Ochratoxin A
CGMCC	China General Microbiological Culture
IARC	International Agency for Research on Cancer Collection Center
ROS	Reactive oxygen species
SOD	Superoxide Dismutase
CAT	Catalase
MDA	Malonic dialdehyde
POD	Peroxidase
